# Resuming post living donor liver transplantation in the COVID-19 pandemic: real-life experience, single-center experience

**DOI:** 10.1186/s43066-021-00153-0

**Published:** 2021-12-20

**Authors:** Abeer Awad Abdellatif, Mohamad Sherif Mogawer, Mostafa El- Shazli, Hanaa El-Karaksy, Ayman Salah, Amany Abdel-Maqsod, Mona El-Amir, Mohamed Said, Naglaa Zayed, Karim Hosny, Hadeel Gamal Eldeen, Ayman M. A. Osman, Doaa A. Mansour, Ahmed Nabil, Ahmed Abdel-Ghani, Engy A. Mogahed, Noha A. Yasin

**Affiliations:** 1 Hepatogastroenterology, Liver Transplantation Unit, Internal Medicine Department, Kasr Al-Aini Hospitals, Cairo University, Kasr Al-Aini Street, Cairo, PO: 11451 Egypt; 2 Department of General Surgery, Liver Transplantation Unit, Kasr Al-Ainy Hospital, Faculty of Medicine, Cairo University, Cairo, Egypt; 3 Pediatrics Department, Liver Transplantation Unit, Cairo University, Cairo, Egypt; 4 Endemic Medicine, Hepatogastroenterology, Liver Transplantation Unit, Kasr Al-Ainy Hospital, Faculty of Medicine, Cairo University, Cairo, Egypt; 5 Endemic Medicine Department, Liver Transplantation unit, Kasr Al-Ainy Hospital, Faculty of Medicine, Cairo University, Cairo, Egypt; 6 Endemic Hepatology and Gastroenterology, Liver Transplantation Unit, Kasr Al-Ainy Hospital, Faculty of Medicine, Cairo University, Cairo, Egypt; 7 General & HPB Surgery, Department of General Surgery, Unit of Hepatobiliary Surgery, Liver Transplantation Unit, Kasr Al-Ainy Hospital, Faculty of Medicine, Cairo University, Cairo, Egypt

**Keywords:** COVID-19, Egypt, Liver transplantation, Single-center experience

## Abstract

**Background:**

Solid organ transplantation (SOT) service has been disrupted during the current coronavirus disease 2019 (COVID-19) pandemic, which deferred the service in most centers worldwide. As the pandemic persists, there will be an urgency to identify the best and safest practices for resuming activities as areas re-open. Resuming activity is a difficult issue, in particular, the decision of reopening after a period of slowing down or complete cessation of activities.

**Objectives:**

To share our experience in resuming living donor liver transplantation (LDLT) in the context of the COVID-19 pandemic in the Liver Transplantation Unit of El-Manial Specialized Hospital, Cairo University, Egypt, and to review the obstacles that we have faced.

**Material and methods:**

This study is a single-center study. We resumed LDLT by the 26th of August 2020 after a period of closure from the 1st of March 2020. We have taken a lot of steps in order to prevent COVID-19 transmission among transplant patients and healthcare workers (HCWs).

**Results:**

In our study, we reported three LDLT recipients, once resuming the transplantation till now. All our recipients and donors tested negative for SARS-CoV-2 by nasopharyngeal RT-PCR a day before the transplantation. Unfortunately, one of them developed COVID-19 infection. We managed rapidly to isolate him in a single room, restricting one team of HCWs to deal with him with strict personal protective measures. Finally, the patient improved and was discharged in a good condition. The second patient ran a smooth course apart from FK neurotoxicity which improved with proper management. The third patient experienced a sharp rise in bilirubin and transaminases on day 14 that was attributed to drug toxicity vs. rejection and managed by discontinuing the offending drugs and pulse steroids. In addition, one of our head nurses tested positive for SARS-CoV-2 that was manageable with self-isolation.

**Conclusion:**

Careful patient, donor, personnel screening is mandatory. Adequate supply of personal protective equipments, effective infection control policies, and appropriate administrative modifications are needed for a safe return of LDLT practice.

## Introduction

Since December 2019, the whole world suffers from a novel coronavirus named severe acute respiratory syndrome coronavirus-2 (SARS-CoV-2) that was declared by the World Health Organization (WHO) as a global pandemic on March 11, 2020; since then, the entire health care environment, especially liver transplant (LT) services, has been affected worldwide [1].

Solid organ transplantation (SOT) is facing many challenges that have been created during the COVID-19 pandemic. Therefore, service provision, restructuring outpatient care, careful screening, donor and recipient selection, and performing LT with limited resources should be redesigned in the post-COVID era [2].

As the pandemic persists, there will be an urgency to identify the best and safest practices for resuming activities as areas re-open. In particular, the decision about resuming SOT remains the most challenging [3].

Indeed, the limitation of data and the highly dynamic COVID-19 pandemic makes a high challenge to weigh the risks and benefits of resuming the SOT amidst fluctuating SARS-CoV-2 community transmission [3].

Owing to the long-term immunosuppressive drugs that LT recipients are exposed to, they may be more susceptible to severe COVID-19 infection with a worse prognosis when compared with the general population. The decision to defer the SOT or not, during this pandemic, is still debatable among many countries especially with the continuing increase in the number of COVID patients in those countries [4].

Many centers suggested that transplantation should be deferred, while others recommended performing transplantation under strict infection precautions and after careful risk assessments. Moreover, the decision should rely upon certain considerations, as center capability regarding the availability of beds in the intensive care unit (ICU), ventilators, and available donated blood [5].

There are still limited published data on the experience with SOT during the COVID-19 era.

### Aim of the work

To share our experience in resuming living donor liver transplantation (LDLT) in the context of the COVID-19 pandemic in the Liver Transplantation Unit of El-Manial Specialized Hospital, Cairo University, Egypt, and to report the obstacles we faced.

### Methodology

This study is a single-center study. Oral and written informed consents were obtained from the patients or from their eligible relatives.

We resumed the LDLT program in our unit at El-Manial Specialized Hospital, Cairo University, by the 26th of August 2020 after a period of closure from the 1st of March 2020. We planned to transplant one case every 21 days to ensure the best quality of service as well as patients’ safety.

We have taken a lot of steps in order to prevent COVID-19 transmission among transplant patients and healthcare workers (HCWs) as working in small subgroups and segregating the teams taking care of immediate post-LT patients from pre-LT patients and from other patients. All organ donor teams, transplant providers, and support staff were aware of this risk and have taken appropriate respiratory contact precautions. Our transplant team members were divided into 4 teams which allowed the continuation of services if any team member became exposed to, or infected with COVID-19. We emphasized to our post-transplant patients about preventive measures that should be followed such as frequent hand washing, cleaning frequently touched surfaces, staying away from large crowds, staying away from individuals who are ill, and refraining from travel during this pandemic. Periodical training and auditing were held by the infection control team to ensure the continuous awareness with the strict infection precautions, proper donning and doffing of personal protective equipment (PPE), and also to observe that these measures were followed strictly by all the HCWs.

Measures to minimize infection risk included maintaining physical distance (1–2 m), washing hands frequently (at least 20 s with soap), minimum hospital visits after transplant and regularly disinfecting surfaces, avoiding hand-face contact as much as possible, and avoiding close contact with patients and if contact is unavoidable, the use of an N95 mask and full PPE was emphasized. All HCWs had to wear (at a minimum) a surgical mask in all clinical settings even in regular staff meetings.

All the patients (recipient and donor) were subjected to thorough history taking for the presence of fever or respiratory symptoms, as well as contact and travel history. Lastly, a COVID-19 test was performed as a final step to exclude asymptomatic COVID-19 infection before proceeding to surgery.

During the transplantation surgery and because the donor and recipient were negative for COVID-19, the surgeons did not require additional use of protective wear. However, during aerosol-generating procedures like intubation and extubation, the anesthesiologists were wearing full PPE.

Recipients were nursed strictly in a single room with only one team of nurses till discharge while applying strict standard and droplets precautions. They were monitored closely for the development of infective symptoms and tested for COVID-19 promptly if any symptoms were reported.

Furthermore, we tried to minimize the number of staff contact with the transplanted patient as possible and all non-essential contact was minimized. Periodic testing of HCWs was performed regularly (twice per month) by nasopharyngeal RT-PCR. Active surveillance was ensured in order to prevent viral spread among HCWs.

## Results

First of all, before starting the transplantation process, a key point was to consider the availability of operating theater, medical staff members, and ICU beds, as these resources may be diverted to the care of patients with COVID-19.

All patients, donors, and personnel were provided with surgical masks and supplies to perform hand hygiene at the entrance of the transplant unit. Before transplantation, both living donors and recipients underwent nasopharyngeal swabs for SARS-CoV-2 by RT-PCR, as well as chest computed tomography (CT) scans the day before the transplantation. Only donors and recipients who tested negative were eligible for transplantation. Suspected or confirmed COVID-19 patients were eliminated from the donation/transplantation process.

High-performance PPE, i.e., an N95 or FFP2/FFP3 respirator, a hairnet, a double pair of gloves, a disposable waterproof surgical gown, a face shield or goggles, and work safety clogs all are supplied to all medical staff and HCWs who get in direct contact with the transplanted patient.

From the 26th of August, we transplanted three patients. All our recipients and donors tested negative for SARS-CoV-2 by nasopharyngeal RT-PCR a day before the transplantation. The first one was a 2.5-year-old child who underwent LDLT for Crigler–Najjar syndrome type I on 26/8/2020. His donor was his 35-year-old father who was medically free. On day 8 post-transplant the patient started to develop fever reaching 38.5°C. Abdominal ultrasound revealed 300 cc of clear appearing subphrenic collection secondary to a minimal biliary leak, for which a pigtail was inserted guided by ultrasonography. Culture and sensitivity of the drained fluid revealed no bacterial growth. Few days later the collection totally resolved while a low-grade fever persisted. On day 11, the patient developed high-grade fever (40°C), tachycardia (130 b/min), and tachypnea (RR=30) for which he was readmitted to the ICU, a nasopharyngeal swab for SARS-CoV-2 was done by RT-PCR and proved positive; CT chest was normal. We managed rapidly by isolating him in a single room, restricting one team of HCWs to deal with him with strict personal protective measures. Supportive treatment was described, fever resolved and the patient ran a smooth course after that (Table 1).

**Table 1:** Laboratory findings of the first patient

Date	Day 1 post-transplant	On discharge	Normal value
TLC	11.9	8.1	4–11 × 10^3^/mm^3^
Lymphocyte	12.8	50	20–45
Hb	12	9.6	12–15 g/dl
PLT	126	382	150–450 × 10^9^/L
CRP	29.6	29	Up to 5 mg/l
Urea	30	18	7–50 g/dl
Creatinine	0.5	0.3	0.60–1.30 mg/dl
Na	137	136	136–145 mmol/l
K	4.1	4.2	3.5–5.1 mmol/l
AST	1104	70	Up to 32 u/l
ALT	1029	39	Up to 35 u/l
Albumin	3.8	2.8	3.5–5.2 g/dl
T. Bilirubin	5	0.6	0.3–1.2 mg/dl
D. Bilirubin	2.5	0.18	0.1–0.3 mg/dl
GGT	37	221	Up to 35 u/l
ALP	32	398	30–120 u/l
PC%	35	86	100%
INR	2.2	1.1	1

*TLC* total leucocytic count, *Hb* hemoglobin, *PLT* platelet count, *CRP* C-reactive protein, *Na* sodium, *K* potassium, *AST* aspartate transaminase, *ALT* alanine transaminase, *T. Bilirubin* total bilirubin, *D. Bilirubin* direct bilirubin, *GGT* gamma-glutamyl transferase, *ALP* alkaline phosphatase, *PC%* prothrombin concentration, *INR* international normalized ratio

The second patient was a 33-year-old male patient who underwent LDLT at 27/9/2020 for cryptogenic liver cirrhosis, repeated attacks of spontaneous bacterial peritonitis (SBP), MELD 23, CHILD 11, the patient was not diabetic or hypertensive, his donor was his 37-year-old-brother who was medically free. On day 5 post-transplantation, the patient developed agitation which was considered most likely to be Tacrolimus neurotoxicity (he was on 2 mg orally twice daily; trough level was 2.6 ng/ml). Tacrolimus was discontinued together with modification of his immunosuppression regimen resulting in marvelous improvement, followed by complete resolution of his agitation. The patient was maintained on prednisone 20 mg orally twice daily and mycophenolate mofetil 1500 mg orally twice daily. He is doing fine till the time of writing the manuscript (Table 2).

**Table 2:** Laboratory findings of the second patient

Date	Day 1 post-transplant	On discharge	Normal value
TLC	7.1	8.7	4–11 × 10^3^/cmm
Lymphocyte	1050	1110	20–45
Hb	9.1	8.2	12–15 g/dl
PLT	26	276	150–450 × 10^9^/L
CRP	5	43	Up to 5 mg/l
Urea	175	62	7–50 g/dl
Creatinine	1.3	1.1	0.60–1.30 mg/dl
Na	139	133	136–145 mmol/l
K	4.2	4.3	3.5–5.1 mmol/l
AST	19	32	Up to 32 u/l
ALT	17	44	Up to 35 u/l
Albumin	2.4	3	3.5–5.2 g/dl
T. Bilirubin	1.5	1.1	0.3–1.2 mg/dl
D. Bilirubin	1.1	0.45	0.1–0.3 mg/dL
GGT	58	173	Up to 35 u/l
ALP	60	127	30–120 u/l
PC%	89%	66%	100%
INR	1.12	1.3	1

*TLC* total leucocytic count, *Hb* hemoglobin, *PLT* platelet count, *CRP* C-reactive protein, *Na* sodium, *K* potassium, *AST* aspartate transaminase, *ALT* alanine transaminase, *T. Bilirubin* total bilirubin, *D. Bilirubin* direct bilirubin, *GGT* gamma-glutamyl transferase, *ALP* alkaline phosphatase, *PC%* prothrombin concentration, *INR* international normalized ratio

The third one was a 10-year-old female patient, who underwent LDLT on 14/10/2020. She had biliary atresia and underwent a Kasai porto-enterostomy at the age of 2 months. Her donor was her 32-year-old mother who was medically free. On day 14, the patient developed sudden onset of deep jaundice with steep rise in liver enzymes. These changes were attributed to drug toxicity (sulphamethoxazole-trimethoprim/fluconazole/amoxacillin-clavulanate) vs. rejection. All suspected medications were discontinued and pulse steroid (solumedrol 10 mg/kg) was administered in a single daily dose for 3 successive days with improvement in total bilirubin and transaminases; steroids were changed to oral form (30 mg/day) with gradual withdrawal (Table 3).

**Table 3:** Laboratory findings of the third patient

Date	Day 1 post-transplant	On discharge	Normal value
TLC	9.5	3.2	4-11 × 10^3^/cmm
Lymphocyte	11	11.9	20–45
Hb	9	9.5	12–15 g/dl
PLT	87	59	150–450 × 10^9^/L
CRP	10.8	4.8	Up to 5 mg/l
Urea	56	48	7–50 g/dl
Creatinine	0.6	0.6	0.60–1.30 mg/dl
Na	136	136	136–145 mmol/l
K	4.4	4.2	3.5–5.1 mmol/l
AST	266	40	Up to 32 u/l
ALT	197	203	Up to 35 u/l
Albumin	3.2	3.5	3.5–5.2 g/dl
T. Bilirubin	13.4	7	0.3–1.2 mg/dl
D. Bilirubin	6.6	4.4	0.1–0.3 mg/dl
GGT	31	341	Up to 35 u/l
ALP	836	216	30–120 u/l
PC%	29	71	100%
INR	2.6	1.26	1

*TLC* total leucocytic count, *Hb* hemoglobin, *PLT* platelet count, *CRP* C-reactive protein, *Na* sodium, *K* potassium, *AST* aspartate transaminase, *ALT* alanine transaminase, *T. Bilirubin* total bilirubin, *D. Bilirubin* direct bilirubin, *GGT* gamma-glutamyl transferase, *ALP* alkaline phosphatase, *PC%* prothrombin concentration, *INR* international normalized ratio

All our donors did well and were discharged by day 3 post-transplant.

Before discharge, all our patients were instructed to follow strict social distancing, facial masks wearing, hand washing, and self-isolation measures.

Despite all these measures, one of our head nurses developed one attack of fever reaching 38 °C with bony pain and headache, nasopharyngeal swab tested positive for SARS-CoV-2 and was managed with self-isolation only.

During our early cases following reopening, we have faced the increased financial burden of transplantation including the shortage of PPE. A major problem we have faced was the difficulty to trace the source COVID-19 infections, as this requires an additional budget, to perform testing for all HCWs who got in contact with the patient.

## Discussion

All healthcare delivery services were significantly disrupted by the global pandemic of COVID-19 [6]. Surely, SOT patients are the most vulnerable group subjected to severe infection, morbidity, and mortality. They also require a high level of care through pre-transplant evaluation, transplant surgery, and post-transplant management [6, 7].

Most of the organ transplantation centers worldwide have postponed all elective organ transplantation, and now we are in the process of resuming SOT. COVID-19 pandemic has created unprecedented circumstances and unique challenges for resuming SOT worldwide. Being a highly dynamic pandemic, our understanding continues to evolve. It remains difficult to provide strong unique recommendations given the paucity of robust data to inform guidance.

Being on immunosuppressive medications, the post-transplant patients are considered at high risk for COVID-19 infection, therefore with reopening care, every effort should be taken to protect them from exposure to the virus [6].

In order to prevent possible patient-to-patient and patient-to-personnel transmission, several aspects should be systematically taken into account. Overcrowding should be always avoided and an adequate air change per hour should be maintained [8].

Many transplant centers worldwide developed a COVID-19 donor and recipient clinical screening programs such as Canada, Switzerland, Italy, and Spain. Accordingly, the Japanese Society for Transplantation established a recommendation to screen donors for significant exposure to COVID-19, travel history to high-risk countries, and symptoms including fever and respiratory symptoms together with home or hospital isolation for 14 days prior to intervention in order to avoid COVID-19 exposure for both lung and liver living donors, in cases where transplantation can be postponed for 14 days. Also, the Korean Society for Transplantation published their recommendation on March 13, 2020, for testing both living and deceased donors for SARS-CoV-2 by a nasopharyngeal swab prior to appointment. However, there is still variation in approach to donation between different countries according to the burden of COVID-19 infection and availability of service resources [9].

Preventative strategies and social distancing measures should be reinforced in living donors, especially within 14 days prior to organ donation. Moreover, a high-risk living donor is either because of COVID-19 symptoms or exposure, postponement of organ donation for at least 28 days is a must. American Society of Transplantation recommends delaying the transplant for at least 14 days if the donor is of intermediate risk for COVID-19 such as those with exposure but no symptoms [10], the donor with resolved symptoms more than 28 days prior to organ donation, and with negative testing repeatedly with at least 24 h apart [10].

The aim of this article was to share our experience in resuming the LDLT program in the context of the COVID-19 pandemic and to report the obstacles that faced us.

In our study we reported three LDLT recipients once resuming the transplantation; unfortunately, one of them developed COVID-19 infection. We managed by isolating him in a single room, restricting one team of HCWs to deal with him with full PPE supplies. Finally, the patient improved and was discharged in a reasonable condition. The second patient ran a smooth course apart from FK neurotoxicity that was managed properly. The third patient experienced a sharp rise in bilirubin and transaminases on day 14 that was attributed to drug toxicity vs. rejection and managed by discontinuing the offending drugs and pulse steroids.

Unfortunately, we were unable to trace the source of COVID-19 infection in our first case, due to the lack of accessibility of performing the test to all HCWs. As for most centers, we are also facing the problem of the increased financial burden of transplantation including and the shortage of PPE.

There are no best practices in a pandemic; therefore, managing best practices in a pandemic requires bold decisions and frequent reassessment of rationales [11].

In Wuhan, the COVID-19 pandemic greatly slowed and then stopped organ donation and transplantation, but the decrease in the number of infections has allowed hospitals in Wuhan to carefully resume deceased donor organ donation and transplantation [12].

COVID-19 infection was reported in a 55-month-old girl, 5 months after undergoing liver transplantation; she recovered completely despite the high level of received immunosuppression [13]. Another case report, records living liver donation from a COVID-19 infected donor, the donor was apparently healthy with mild symptoms; lopinavir plus ritonavir were started to the recipient then shifted to hydroxychloroquine due to drug-drug interaction. Fortunately, the result of the serial COVID-19 RT-PCR test via both nasopharyngeal swab and serum was negative. Further information on the pathogenesis and transmissibility of COVID-19 in organ transplantation is still required [14].

Hyo-Lim Hong et al. [14] and Stephen Lagana et al. [15] have reported 2 cases of donor-derived transmission of COVID-19; therefore, a strategy is needed to prevent donor-derived transmission from all potential asymptomatic carriers.

In Italy, out of 17 liver transplanted patients, 2 developed COVID-19 on postoperative days 9 and 22 [16]. On the contrary, a center in China, among six liver transplants performed during COVID-19, no complications were reported [17].

It is still confusing whether the infection source is nosocomial, donor-derived, or just delayed diagnosis of asymptomatic recipients.

Hence, the recommendations for transplantation from donors diagnosed with COVID-19 are prudent, so it is of utmost importance to screen donors for COVID-19 by epidemiological investigations and clinical history for suspected COVID-19 as well as PCR within 3 days of procurement and CT, when feasible [18].

Currently, many SOT centers across the world recommend using CT to screen asymptomatic living donors for COVID-19 in the preoperative evaluation process; however, the American Society of Transplantation is against this issue [6].

For the exclusion of asymptomatic infection in donors, most of the centers have already adopted real-time-PCR and CT scan screening along with serology. However, without complete isolation of the transplant process from cross-contamination and the capability for identification of all asymptomatic COVID-19 patients, the levels of transplantation will not reach their baseline level as it was in the pre-COVID-19 era [6].

Indeed, all the reopening measures should be considered in the context of the pandemic where the possibility of a second peak or even further peaks is still possible.

Furthermore, every effort should be made to maintain one full set of transplant armamentaria in a COVID-19 area, when still in place, in order to perform all SOT in an isolated clean environment with minimization of the risk of COVID-19 transmission.

Finally, strategic planning and coordination will be needed to ensure the robust enrolment of SOT patients in ongoing clinical trials once routine care in the COVID-19 era is reopened.

## Conclusion

In this context during the COVID-19 pandemic, resuming transplantation under the umbrella of established infection control measures is a must. The pandemic has highlighted the utmost importance of working as a team and to share knowledge and experience for the benefit of patients. Indeed, many kinds of research for COVID-19 infection whether regarding outcomes, predictive diagnostics, and management strategies including the optimal approach for resuming SOT are largely needed.

## Recommendations

- We recommend COVID-19 screening for both recipient and donor prior to transplantation.
- We recommend ensuring the availability of ICU beds, ventilators, and available donated blood prior to transplantation.
- We recommend strict prevention measures for post-transplant patients including frequent handwashing, cleaning frequently touched surfaces, staying away from large crowds, staying away from individuals who are ill, and not to travel during this pandemic.
- Reopening is considered in the context of the possibility of a second peak pandemic.

## Data Availability

Not applicable

## Abbreviations

CHILD: Child-Pugh score
CT: Chest computed tomography
HCWs: Healthcare workers
ICU: Intensive care unit
LDLT: Living donor liver transplantation
LT: Liver transplant
MELD: Model for end-stage liver disease
PPE: Personal protective equipment
RT-PCR: Reverse transcription-polymerase chain reaction
SARS-CoV-2: Severe acute respiratory syndrome coronavirus-2
SOT: Solid organ transplantation
WHO: World Health Organization
